# Enhancement of Piezoelectric Properties in Electrospun PVDF Nanofiber Membranes via In Situ Doping with ZnO or BaTiO_3_

**DOI:** 10.3390/mi17010012

**Published:** 2025-12-23

**Authors:** Zhizhao Ouyang, Jinghua Lin, Renhao Rao, Guoqin Huang, Gaofeng Zheng, Changcai Cui

**Affiliations:** 1 Institute of Manufacturing Engineering, Huaqiao University, Xiamen 361021, China; ouyangzz@stu.hqu.edu.cn (Z.O.);; 2 Pen-Tung Sah Institute of Micro-Nano Science and Technology, Xiamen University, Xiamen 361102, China; 3 College of Metrology Measurement and Instrument, China Jiliang University, Hangzhou 310018, China

**Keywords:** electrospinning, in situ doping, Polyvinylidene fluoride (PVDF), zinc oxide (ZnO), piezoelectric properties

## Abstract

High-performance piezoelectric poly(vinylidene fluoride) (PVDF) has great application potential in the field of microsensors, but achieving efficient polarization remains a challenge. Here, the in situ doping electrospinning technique is employed to enhance the piezoelectric properties by introducing a single dose of zinc oxide (ZnO) or barium titanate (BaTiO_3_,BTO) dopants. The effects of key processing parameters on the morphology of nanofiber membranes were systematically investigated. In addition, the influence of zinc oxide (ZnO) or barium titanate (BTO) dopant concentrations on the piezoelectric properties of PVDF was examined. The microstructure, electrical performance, and β-phase content of the composite membranes were characterized. Results indicate that the composite film with a doping formulation of 16 wt% PVDF and 10 wt% ZnO exhibits optimal overall performance: the β-phase content of PVDF reaches 52.8%, and the output voltage reaches 1.5 V, which is 2.5 times higher than that of the undoped PVDF nanofiber membranes. This study provides an effective doping strategy for the fabrication of high-performance piezoelectric nanofiber membranes.

## 1. Introduction

At present, flexible piezoelectric sensors are attracting increasing attention due to their fast response and low energy consumption, and they show great application potential in many fields. The key to flexible sensors lies in the piezoelectric materials, in which the efficient polarization preparation remains a challenge. The high output of flexible piezoelectric materials with high dielectric constants is the key to the application of micro/nano flexible sensors. Polyvinylidene fluoride (PVDF) is a polar crystalline polymer material with high dielectric properties [1,2,3,4,5]. There are five different crystal forms for PVDF, among them the β crystal form has a fully transverse (TTTT) conformation and the best piezoelectric performance [6]. The transformation from the α phase to the β phase is crucial for the practical use of flexible sensors with high piezoelectric performance and offers broad application prospects. Therefore, it is extremely urgent to improve the performance of PVDF membranes.

To meet the performance requirements for flexible piezoelectric sensors, PVDF materials are generally modified by several methods, such as chemical crosslinking, blending, and compositing with nanomaterials. Compared with chemical modification or nanomaterial compositing methods, the polymer blending method is an effective way to regulate the structure and modification of polymers, and to prepare high-performance materials in an economical and efficient manner [7,8,9,10,11,12]. Therefore, polymer blending is an effective approach for preparing flexible piezoelectric sensors [13]. Compared with pure PVDF material, PVDF blend materials have excellent piezoelectric, dielectric, and mechanical properties. Liu et al. [14] prepared PVDF/PAN composite membranes by electrospinning technology and found that the β phase content of the composite membranes was 83.4%, with a tensile elongation at break and tensile strength of 26% and 7 MPa, respectively. Mu et al. [15] first proposed to mechanically blend PVDF/PAN powder with silicone rubber to prepare pressure sensors. The maximum output voltage of the sensor was 49 V, and the current and voltage sensitivities were 0.174 nA/kPa and 42 mV/kPa, respectively. It still showed high stability after 10,000 cycles. Zhao et al. [16] prepared PVDF/PMMA blend membranes by biaxial stretching. It was found that when the addition amount of PMMA was 30 wt% and the stretching ratio was 2 × 2, the β-phase content of the composite film was the highest, reaching 93%. Mishra et al. [17] prepared PVDF/PLA blend materials by solution casting. After adding PLA, the β-phase content in PVDF increased, confirming the successful interaction between PVDF and PLA at their interface, which simultaneously promoted interfacial polarization and improved the dielectric properties of the material.

In this study, electrospinning technology was employed to blend ZnO and BTO into the PVDF solution to fabricate composite nanofibrous membranes. The reaction mechanism of the interaction between ZnO and PVDF involves physical mixing, chemical bonding, and possible interfacial interactions. Recent studies by relevant scholars have shown that inorganic fillers have significant potential in enhancing the performance of polymer composites. Studies on the atomic level of BaTiO_3_ by Aisulu U. et al. provided a comprehensive review, highlighting its wide applicability in energy-related applications [18]. Eloisa et al. discussed the synthesis and application of ZnO nanostructures and electrospun ZnO composite polymers, demonstrating their effectiveness in sustainability and biomedical fields [19]. This progress highlights the rationality of choosing ZnO and BTO as dopants to enhance the piezoelectric properties of PVDF nanofiber membranes. The incorporation of ZnO can accelerate the crystallization behaviors of PVDF, promoting the formation of the β-phase crystal structure [20], enhance the crystallization kinetics of PVDF, providing a simpler and easier way for the formation of the β-phase crystal structure [21], and provide a strong inducing effect to stretch PVDF molecular chains into the β-phase crystal structure during the crystallization process. The reaction mechanism between BTO and PVDF mainly relies on physical mixing and interfacial interactions [22]. The BTO particles act as heterogeneous nucleating agents, providing more crystallization sites for the PVDF molecular chains, which are conducive to the formation of the β phase crystal structure [23]. The incorporation of BTO may have changed the crystallization kinetics process of PVDF, affecting the movement rate and crystallization rate of the PVDF molecular chains, making the formation of the β phase crystal structure easier and faster; the electric field generated by the BTO particles may help the rearrangement and orientation of the PVDF molecular chains, thereby promoting the formation of the β phase crystal structure.

An in situ doping process is presented in this manuscript for achieving highly efficient output from high-performance piezoelectric PVDF nanofibrous membranes. The dopants of zinc oxide (ZnO) and barium titanate (BaTiO_3_, BTO) are induced into the piezoelectric nanofiber during the electrospinning process. 

## 2. Materials and Methods

### 2.1. Materials

Polyvinylidene fluoride (PVDF, *molecular weight:M*w = 4.0 × 10^5^, *purity*: ω > 99.5%), N,N-dimethylformamide (DMF, purity: ω > 99.0%), barium titanate (BaTiO_3_, purity: ω > 99.5%, Powder < 3 μm), zinc oxide aqueous solution (ZnO·H_2_O), isopropyl alcohol, acetone, were all purchased from Guangxin Industrial Products (Chuzhou, China).

### 2.2. Optimization of Electrospinning Processing Parameters

Electrospinning is a highly promising nanofiber fabrication technology capable of producing nanofibrous materials with high specific surface areas, exceptional porosity, and distinctive microstructures. These characteristics endow it with significant application potential across a variety of cutting-edge research fields. However, electrospinning represents a nonlinear dynamic system, entangled in complex fluid dynamics and multi-physics field interactions of electrical, fluidic, and thermal, wherein the optimization of processing parameters occupies a pivotal position in the advancement of this technical framework. The electric field intensity serves as the primary driving force for the formation of jets from polymer solutions or melts. Its magnitude and distribution directly affect the initial shape, stretching degree, and movement trajectory of the jets. When the intensity is too low, the jets cannot obtain sufficient energy to overcome surface tension, resulting in difficulties in the continuous progress of the spinning process; while an excessively high electric field intensity may cause unstable breakage of the jets, leading to defects such as spherical fibers. A shorter spinning distance may prevent the solvent from fully evaporating, causing the fibers to stick together on the collection device, whereas an excessively long spinning distance will increase the flight time of the jets, potentially resulting in uneven distribution of fiber diameters.

**Figure 1:**
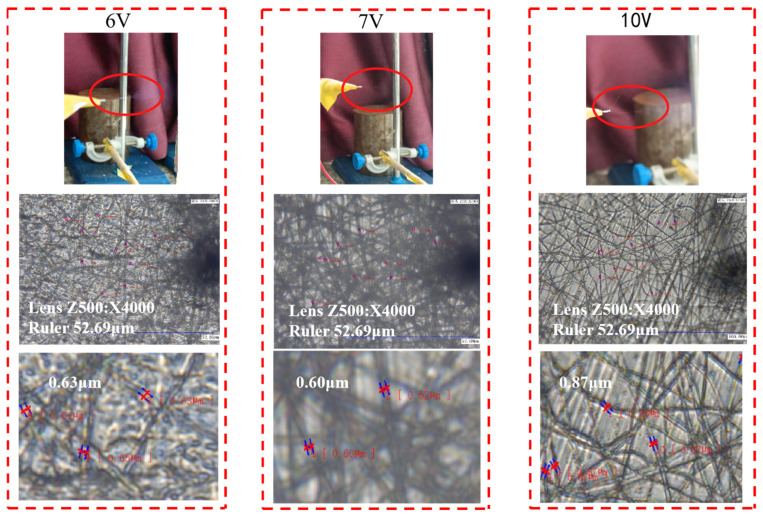
Polyvinylidene fluoride (PVDF) nanofibers prepared under the same conditions with different applied voltages.

**Figure 2:**
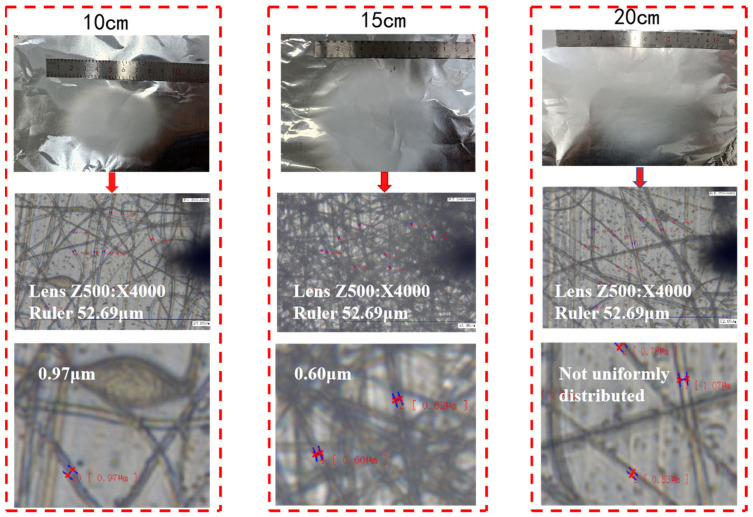
Under the same conditions, PVDF nanofibers prepared with different collection distances.

In this study, we conducted a simple investigation of the external voltage and collection distance during the electrospinning process using the same concentration of PVDF. The results are shown in Figure 1 and Figure 2.

From Figure 1 and Figure 2, it can be seen that when the applied voltage and collection distance are different, the prepared nanofibers will also vary. As shown in Figure 2, when the collection distance is 10 cm, the collection distance is too short, the nanofibers are deposited too densely, and the jetting time is too short, resulting in the formation of bead-like fibers. The number of fibers is small, and the diameters are relatively large. When the collection distance is too long, the jetting time is also longer, the diameters of the fibers are not uniformly distributed and the fibers are more scattered on the collection device, which is not conducive to subsequent processing. Therefore, the optimized process parameters are an applied voltage of 7 kV and a collection distance of 15 cm.

### 2.3. Preparation of Polyvinylidene Fluoride (PVDF) and Composite PVDF Nanofiber Membranes

PVDF nanofiber membranes were prepared using an electrospinning device. Different concentrations of PVDF solutions were prepared, as shown in Table 1. The DMF/acetone mass ratio was 4:3. After preparing the PVDF base solution, the ZnO-doped solution was slowly added to the PVDF solution. Then, the mixture was subjected to magnetic stirring at a speed of 400 revolutions per minute in a constant temperature water bath at 75 °C, and ultrasonic treatment was carried out for 5 h. During the electrospinning process, the voltage was 7 kilovolts, the liquid supply rate was 5 microliters per minute, the collection distance was 15 centimeters, the temperature was 29.6 °C, and the humidity was 77.6%. The BTO-doped solution was treated in a similar manner.

Under the aforementioned preparation conditions, the optimal concentration of the PVDF nanofibrous membranes was determined through characterization and testing. To investigate the extent of the influence of different dopants on the performance of the PVDF nanofibrous membranes, different doping concentrations were prepared, as shown in Table 2. All preparation conditions were the same as the above. The concentrations of the doping solutions (ZnO, BTO) are all the proportions of each solution in the total mixed solution mass.

### 2.4. Characterization and Testing

The morphology and structure of the nanofiber membranes were characterized by scanning electron microscopy (SEM) [24]. The content of the β phase in the nanofiber membranes was measured using a Fourier infrared spectrometer [25]. The scanning range was (400–4000) cm^−1^, with 32 scans and a resolution of 4 cm^−1^. The piezoelectric performance output of the packaged flexible piezoelectric sensor was tested using a self-assembled piezoelectric testing system. A 10 Hz sinusoidal wave signal was generated by a signal generator (Agilent-33522A, Keysight Technologies, Santa Rosa, CA, USA), and it was input into the spring testing machine through a power amplifier (PA-1200, MONACOR, Bremen, Germany) to generate periodic vibrations. The flexible pressure sensor was struck to produce deformation. The voltage output signals generated during the striking process were recorded by an oscilloscope (DPO-3012, Tektronix, Beaverton, OR, USA) and a digital source meter (KEITHLEY-2400, Cleveland, OH, USA), respectively.

## 3. Results

### 3.1. Characterization and Testing of PVDF Nanofibrous Membranes

**Figure 3:**
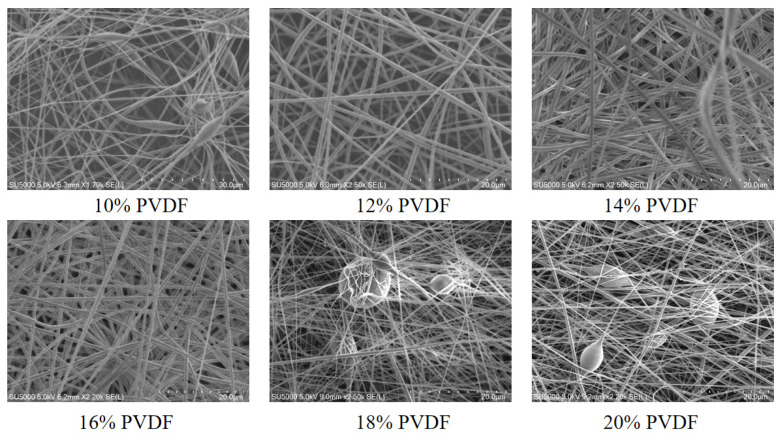
Surface morphology images of nanofibers prepared from different concentrations of PVDF.

For the PVDF nanofiber membranes obtained, the surface morphology was observed using a scanning electron microscope to study the influence of solution concentration on the morphology of the nanofibers. The results are shown in Figure 3. From Figure 3, it can be seen that when the concentration is 10%, the surface morphology of the prepared PVDF nanofiber film contains bead-like structures. When the concentration is 12%, the bead-like nanofibers disappear, but the fiber diameters are not uniform. However, when the concentration is 14%, the fiber surface is smooth, and the fiber diameter distribution is relatively uniform. When the concentration is 16%, the surface morphology is not much different from that of the 14% PVDF nanofibers. Based on the average diameter of the fibers, the average fiber diameter of 14% PVDF can be calculated as 5.30 μm, while the average fiber diameter of 16% PVDF is even smaller, at 4.77 μm. Moreover, as the solution concentration increases, the number of fibers also increases to a certain extent. The reason for this phenomenon lies in the solution concentration. The solution concentration is the decisive factor affecting the entanglement of the molecular chains in the solution. Insufficient molecular chain entanglement in the solution, uneven stretching forces, and inconsistent orientation of the molecular chains lead to the generation of bead-like fibers. The size and quantity of the fibers also have a certain correlation with the solution concentration. As the solution concentration increases, more polymer molecules are brought into the spinning process, thereby increasing the number of fibers.

**Figure 4:**
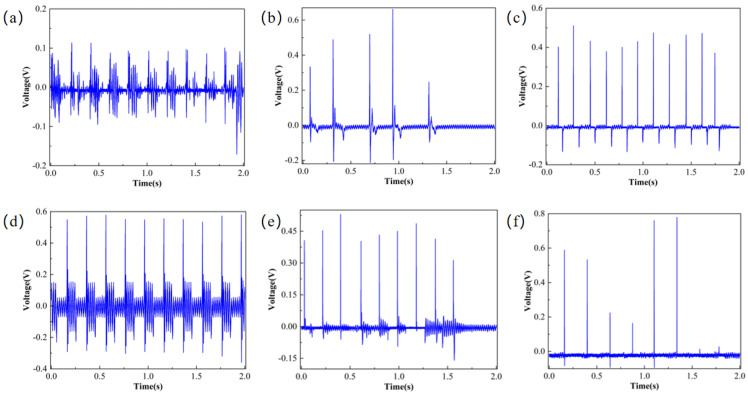
Piezoelectric responses of nanofibers prepared from different concentrations of PVDF; (**a**) 10% PVDF, (**b**) 12% PVDF, (**c**) 14% PVDF, (**d**) 16% PVDF, (**e**) 18% PVDF, (**f**) 20% PVDF.

After characterizing the surface morphology of the PVDF nanofibrous membranes, the nanofibrous membranes were then fabricated into a simple sensor in a “sandwich” structure. A simple testing platform was composed using a spring force gauge, a signal generator, a power amplifier, and an oscilloscope. The results are shown in Figure 4. The 10% PVDF nanofibrous membranes voltage exhibited a state of oscillation and fluctuation, and some data points deviated from the stable state. The 12% and 20% PVDF nanofibers showed several extremely high voltages, but the voltage stability was relatively low. The 14%, 16%, and 18% PVDF nanofibrous membranes voltages had stronger stability, among which the voltage of 16% PVDF averaged at 0.6 V, which was slightly larger than those of 14% and 18%.

Based on the surface morphology and voltage performance test results, a PVDF solution with a concentration of 16% was used as the base, and different concentrations of ZnO solution and BTO solution were doped into it. This study deeply explored the influence of the doped solutions on the voltage performance of the PVDF nanofibrous membranes.

### 3.2. Characterization and Testing of PVDF/ZnO Composite Film

**Figure 5:**
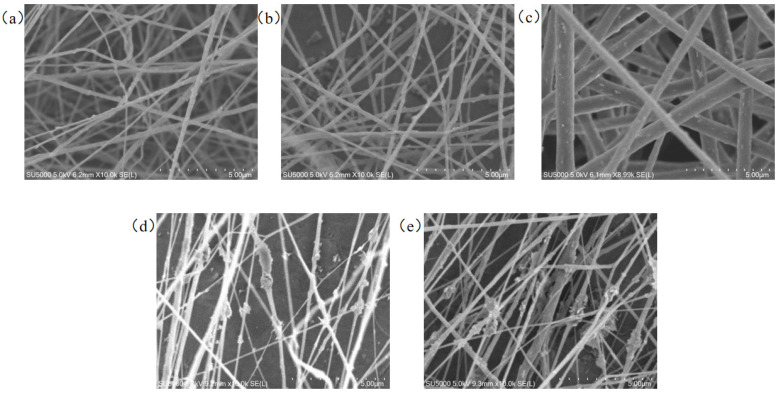
SEM images of composite nanofibers prepared by doping solutions of different concentrations of ZnO; (**a**) 16% PVDF/1% ZnO, (**b**) 16% PVDF/5% ZnO, (**c**) 16% PVDF/10% ZnO, (**d**) 16% PVDF/15% ZnO, (**e**) 16% PVDF/20% ZnO.

ZnO solutions of different concentrations (Table 2) were mixed with the 16% PVDF solution to prepare composite solutions. PVDF composite nanofiber membranes were then fabricated by electrospinning, and their surface morphology is shown in Figure 5. The SEM images show that the composite nanofibers doped with 1 wt% ZnO have non-uniform diameters, and the ZnO particles are not clearly visible on the fiber surface. In the composite nanofibers doped with 5% ZnO, there is a phenomenon of ZnO particle aggregation. In contrast, for the composite nanofibers doped with 10 wt% ZnO, the ZnO particles are clearly and uniformly attached to the fibers, and the fiber diameters are more uniform, leading to improved morphology and performance. The reason for the aggregation of ZnO particles may be the self-aggregation mechanism of ZnO particles. When the ZnO particles are refined to the nanoscale, their surface accumulates a large amount of positive and negative charges. The accumulation of charges leads to an increase in the electrostatic attraction between the nanoparticles, making it easier to aggregate. On the other hand, when the ZnO concentration is low, there are relatively fewer ZnO particles, but the factors such as surface charge and surface energy of each particle still cause them to easily aggregate. The limited number of ZnO particles may lead to aggregation into a few large clusters. When the concentration increases, the number of particles also increases, and the interactions between particles become more complex, the interaction between particles becomes more complex, which may lead to more obvious adhesion phenomena.

**Figure 6:**
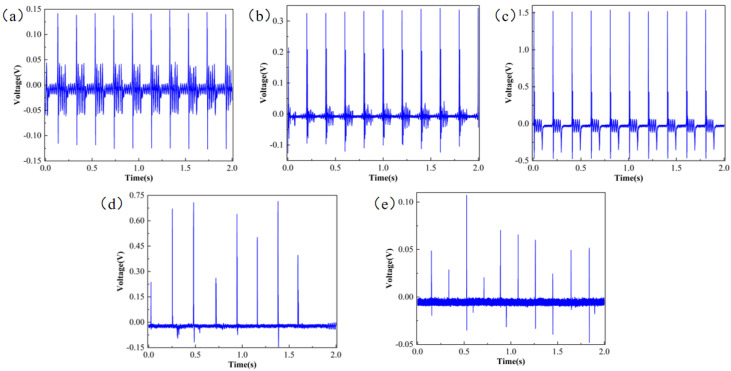
Voltage responses of ZnO-doped nanofiber membranes at different concentrations; (**a**) 16% PVDF/1% ZnO, (**b**) 16% PVDF/5% ZnO, (**c**) 16% PVDF/10% ZnO, (**d**) 16% PVDF/15% ZnO, (**e**) 16% PVDF/20% ZnO.

The output voltage results are shown in Figure 6. The output voltages of the composite fibers containing 1% ZnO, 5% ZnO, and 10% ZnO are stable and good. The output voltage of the 10% ZnO composite fiber reaches 1.5 V, which is more stable than that of the 10% ZnO composite fiber.

**Figure 7:**
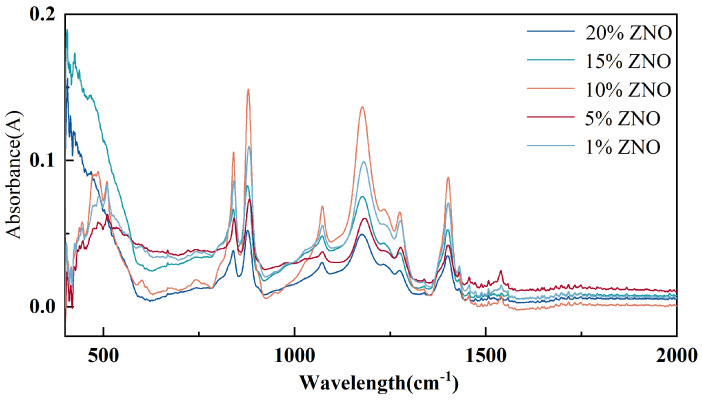
Infrared spectra of composite nanofibers doped with different concentrations of ZnO.

The content of the β phase in the composite film was measured by Fourier infrared spectroscopy. The scanning range was (400–4000) cm^−1^, the scanning times were 32, and the resolution was 4 cm^−1^. The test results are shown in Figure 7. The absorbance spectra of PVDF/ZnO composite nanofibers with different ZnO concentrations fluctuate with wavelength. The curve of high-concentration ZnO had a larger amplitude of peak and valley compared to that of low-concentration ZnO. The characteristic peak at the wavelength of 840 cm^−1^ corresponded to the α phase, and the characteristic peak at 878 cm^−1^ corresponded to the β phase. The content of the piezoelectric β phase was quantified using the Lambert–Beer formula:

In the formula: Aα and Aβ represent the absorbance at 840 cm^−1^ and 878 cm^−1^, respectively. The calculation results are shown in Table 3. The sample with the highest β-phase content is the one doped with 10% ZnO, reaching 52.8%, which is higher than that of pure PVDF at 52.4%.

By characterizing the surface morphology of the composite film nanofibers, testing the piezoelectric properties of the composite film, and calculating the content of the β phase in the composite film, it was concluded that the composite solution doped with 10% ZnO, when used to prepare the composite film, exhibited better piezoelectric properties, superior to the pure PVDF film. The particles attached to the fiber surface were evenly distributed, the fiber diameter was uniform, and the voltage output reached 1.5 V, which was 2.5 times higher than that of the pure PVDF, and the content of the β phase was also higher. The electrical output results are used to demonstrate relative performance trends among different doped membranes rather than statistically optimized absolute values.

### 3.3. Characterization and Testing of PVDF/BTO Composite Film

**Figure 8:**
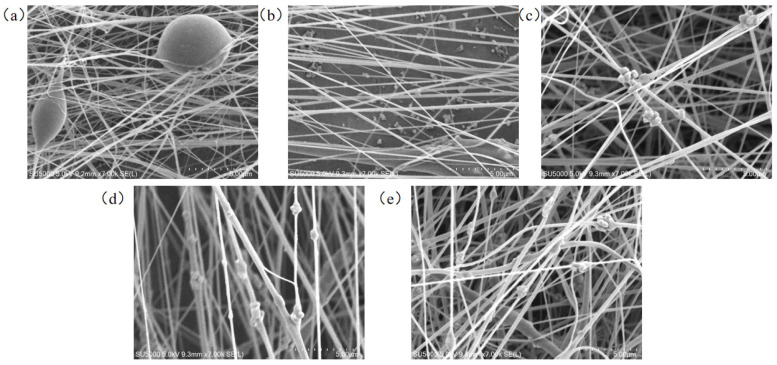
SEM images of composite nanofibers prepared by doping different concentrations of BTO solution; (**a**) 16% PVDF/1% BTO, (**b**) 16% PVDF/5% BTO, (**c**) 16% PVDF/10% BTO, (**d**) 16% PVDF/15% BTO, (**e**) 16% PVDF/20% BTO.

BTO solutions with different concentrations (Table 2) were mixed with the 16 wt% PVDF solution to prepare composite solutions. PVDF composite nanofiber membranes were then fabricated by electrospinning, and their surface morphology is shown in Figure 8. The SEM images show that the nanofibers doped with 1 wt% BTO contain abnormal bead-like and spherical structures. In contrast, for the nanofibers doped with 5 wt% BTO, no obvious particle adhesion is observed, and the spinning jet tends to drip and leak at the collector, likely due to the low BTO concentration and poor mixing with PVDF. The possible reason for this is that the concentration of the doped BTO solution is too low and has not been mixed evenly with PVDF. As the BTO concentration increases, the density of BTO particles between the nanofibers also increases. For the 15 wt% and 20 wt% BTO samples, more BTO particles can be observed adhering to the fibers, and the fiber morphology also shows noticeable differences due to the influence of the BTO particles.

**Figure 9:**
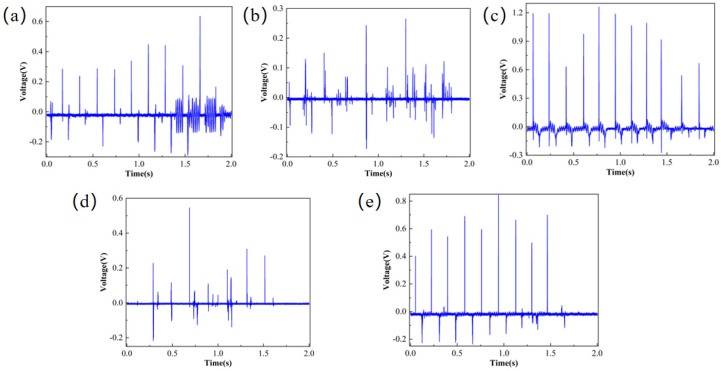
Voltage responses of nanofiber membranes doped with different concentrations of BTO; (**a**) 16% PVDF/1% BTO, (**b**) 16% PVDF/5% BTO, (**c**) 16% PVDF/10% BTO, (**d**) 16% PVDF/15% BTO, (**e**) 16% PVDF/20% BTO.

The piezoelectric properties of the PVDF/BTO composite film were tested using the test platform, and the results are shown in Figure 9. Figure 9a shows frequent voltage fluctuations with a large amplitude, presenting an irregular fluctuation pattern; Figure 9b shows that there are fewer voltage fluctuations, and the changes are relatively smooth. The reason for this phenomenon may be that the concentration of the BTO solution is low, and few BTO particles adhere to the fibers, destroying the original morphology of the fibers. As the concentration of the BTO solution increases, the voltage changes are more obvious. The voltage of the 10% BTO-doped film is about 1.2 V, showing a stable state. However, a higher BTO concentration does not necessarily result in better performance. An excessively high concentration of BTO will cause the BTO particles to be unevenly dispersed in the PVDF matrix, resulting in agglomeration, and will instead reduce the performance of the composite film. For example, the voltage effect shown by the 15% and 20% BTO solutions is worse than that of the 10% BTO solution.

**Figure 10:**
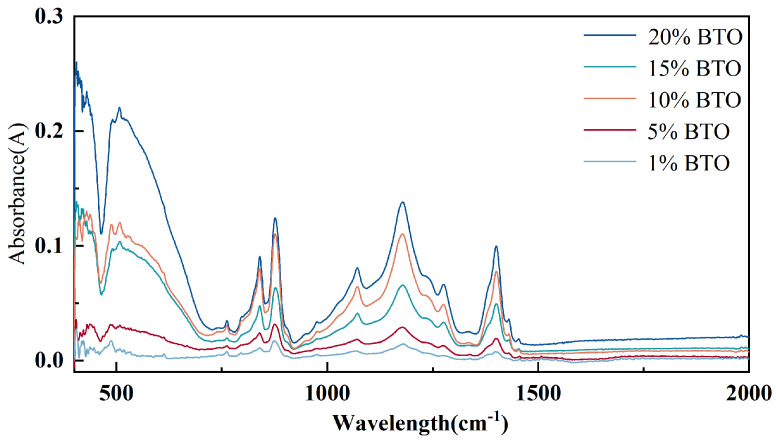
Infrared spectra of composite nanofibers doped with different concentrations of BTO.

As mentioned above, the content of the β phase in the composite film was measured and calculated using Fourier infrared spectroscopy. The infrared spectroscopy measurement is shown in Figure 10. As the concentration of BTO increased, the intensity of the absorption peaks changed. The absorption peak intensity of 20% BTO was relatively high, while that of 1% BTO was relatively low, showing a certain linear relationship. However, the height of the absorption peaks does not indicate the content of the β phase. We used the formula to calculate the content of the β phase; the results are shown in Table 4. It can be seen that the content of the β phase of 1% BTO is 52.8%, which is slightly higher than that of 20% BTO.

## 4. Conclusions

Based on the characterization of the surface morphology and output voltage of PVDF nanofibers, a 16 wt% PVDF solution was determined to be the optimal concentration for preparing nanofibers. Using this as the base, different concentrations of doped composite nanofiber membranes were prepared. The surface morphology, β-phase content, and output voltage of the composite membranes were characterized and tested. By comparing the results, it was found that the composite solution doped with 10% ZnO had an output voltage of 1.5 V, which was the highest and most stable among all the composite membranes, and the β-phase content was 52.8%, which was also the highest among all the composite membranes. Compared with pure PVDF, the β-phase content of the composite film only slightly increased, but the output voltage increased by 150% compared with pure PVDF. The reason for this result is that the voltage output is not solely determined by the β phase, but is also influenced by factors such as fiber morphology, effective dielectric constant, and interface polarization. Similarly, the output voltage of the BTO-doped composite film increased by about 25%. Therefore, a 16 wt% PVDF solution doped with 10 wt% ZnO is considered the optimal composition for preparing PVDF composite membranes.

## Figures and Tables

**Table 1:** Ratio of different concentrations of Polyvinylidene fluoride (PVDF) solutions.

Solution	Concentration
PVDF	10%
PVDF	12%
PVDF	14%
PVDF	16%
PVDF	18%
PVDF	20%

**Table 2:** Solutions with different concentrations of doping (the impurity content ratio is the total mass of the mixed solution).

Solution	Concentration
ZnO	1%
ZnO	5%
ZnO	10%
ZnO	15%
ZnO	20%
BTO	1%
BTO	5%
BTO	10%
BTO	15%
BTO	20%

**Table 3:** Content of β phase in nanomembranes.

Film Type	F_(β)_
16% PVDF	52.4%
16% PVDF/1% ZnO	50.1%
16% PVDF/5% ZnO	48.5%
16% PVDF/10% ZnO	52.8%
16% PVDF/15% ZnO	49.5%
16% PVDF/20% ZnO	51.7%

**Table 4:** Content of β phase in nanofibrous membranes.

Film Type	F_(β)_
16% PVDF	52.4%
16% PVDF/1% BTO	52.8%
16% PVDF/5% BTO	49.9%
16% PVDF/10% BTO	51.4%
16% PVDF/15% BTO	50.9%
16% PVDF/20% BTO	51.2%

## Data Availability

The data presented in this study are available on request from the corresponding author.
